# Supplementary material for: Relationships of Gut Microbiota Composition, Short-Chain Fatty Acids and Polyamines with the Pathological Response to Neoadjuvant Radiochemotherapy in Colorectal Cancer Patients
Source: Int J Mol Sci. 2021 Sep 2;22(17):9549. doi: 10.3390/ijms22179549 (PMC8430739; doi:10.3390/ijms22179549)
Supplement: Supplementary file 1 [file ijms-22-09549-s001.zip › ijms-1366570-supplementary.pdf]

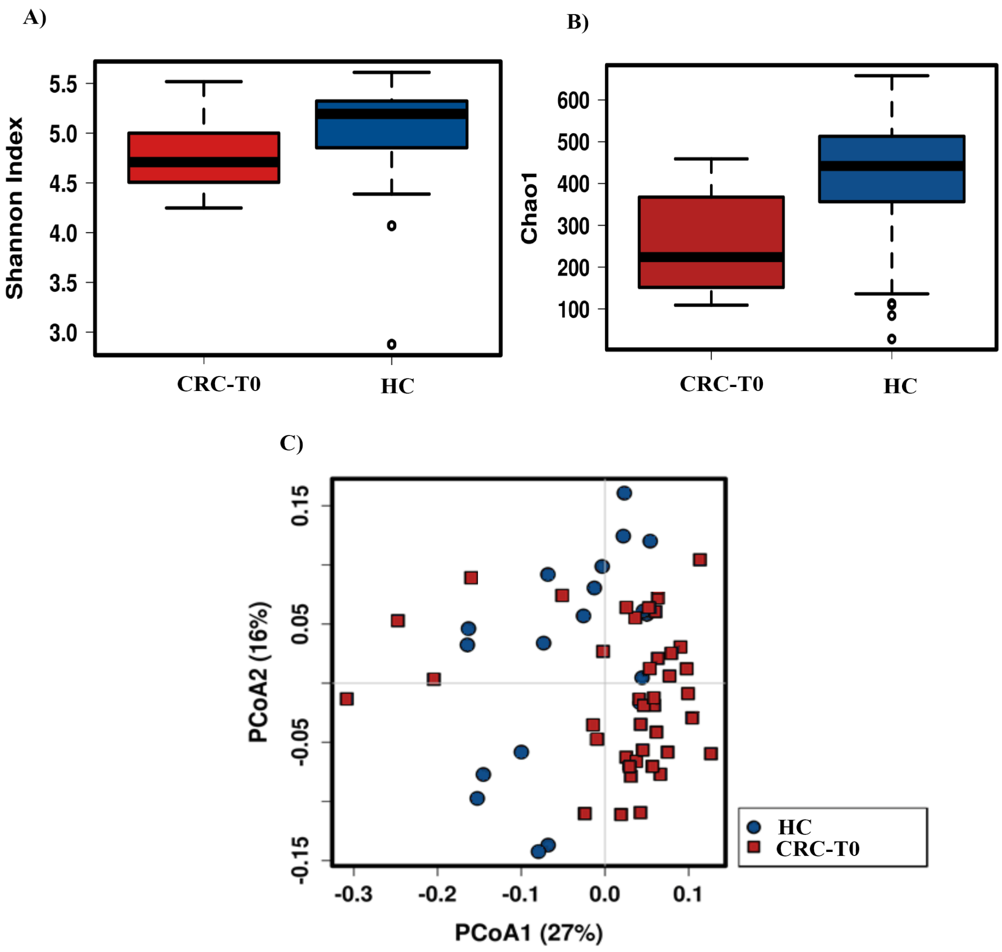

Figure S1. Comparison of alpha and beta diversity among CRC patients at baseline (CRC-T0) and healthy controls (HC). **(A)** Shannon index (p=0.026); **(B)** Chao1 index (p=0.001); **(C)** Principal component plot based on Bray-Curtis distance matrix from patients with CRC and healthy controls using Bray-Curtis dissimilarity index at genus-level (p=0.0001). The first two coordinates are plotted with the percentage of variability explained indicated on the axis.
